# Infarct Laterality Patterns in Relation to A1 Segment Hypoplasia/Aplasia According to Etiological Subtype

**DOI:** 10.3390/brainsci16050486

**Published:** 2026-04-30

**Authors:** Junpei Nagasawa, Tatsuhiro Yokoyama, Ryuichi Okamoto, Junya Ebina, Mari Shibukawa, Takehisa Hirayama, Osamu Kano

**Affiliations:** 1 Department of Neurology, Toho University Faculty of Medicine, Tokyo 143-8541, Japan; junpei.nagasawa@med.toho-u.ac.jp (J.N.);; 2 Department of Neurology, Toho University Omori Hospital, 5-21-16 Omorinishi, Ota-ku, Tokyo 143-8541, Japan

**Keywords:** Circle of Willis, A1 segment hypoplasia, cerebral infarction, embolic stroke of undetermined source, lacunar infarction, magnetic resonance angiography, cardiogenic cerebral embolism

## Abstract

**Background:** The Circle of Willis (CoW) is a key collateral pathway that enables communication between the anterior and posterior cerebral circulations. However, anatomical variations in the A1 segment of the anterior cerebral artery, such as hypoplasia or aplasia, can alter hemodynamics and may compromise this collateral function. While incomplete CoW configurations have been linked to aneurysm formation and altered patterns of hemorrhage, their role in the distribution of cerebral infarctions remains controversial. We aimed to explore the association between A1 segment hypoplasia/aplasia and infarct laterality across different etiological subtypes. **Methods:** We retrospectively analyzed patients with unilateral anterior circulation infarction admitted between April 2017 and March 2023. The CoW was assessed by magnetic resonance angiography (MRA). A1 segment hypoplasia was defined as a segment diameter <1 mm, and A1 aplasia was defined as non-visualization on MRA. The side with hypoplasia or aplasia was defined as the minor side, and the contralateral side as dominant. We assessed whether infarction occurred on the minor or dominant side. **Results:** Among 198 patients with unilateral anterior circulation infarction classified as lacunar, cardioembolic stroke (CES), or embolic stroke of undetermined source (ESUS), 30% had A1 hypoplasia or aplasia, with similar prevalence across subtypes. Infarcts occurred on the A1 dominant side in 53% of lacunar, 55% of ESUS, and 75% of CES cases. Although this difference did not reach statistical significance (*p* = 0.43), it should be interpreted with caution given the limited sample size. **Conclusions:** The rates of A1 hypoplasia and aplasia were similar across stroke types. No statistically significant association was identified. The findings remain inconclusive given the limited sample size. These results should be considered exploratory and hypothesis-generating.

## 1. Background

The Circle of Willis (CoW) is a ring-shaped structure consisting of anastomoses of the anterior cerebral artery (ACA), middle cerebral artery, posterior cerebral artery, anterior communicating artery, and posterior communicating artery. The CoW connects the blood flow between the left and right cerebral hemispheres, as well as between the anterior and posterior circulations. The CoW has a high rate of anatomical variations (fenestration, duplication, hypoplasia, or absence of components) [1], and a complete CoW (in which no component is absent or hypoplastic) is only seen in 37.2–48% of individuals [2,3].

The effect of CoW variations on stroke development remains unclear. Several studies have reported that an incomplete CoW is associated with cerebral aneurysms [4,5]. Regarding cerebral hemorrhage, it has been reported that when A1 hypoplasia is present, putaminal hemorrhage is more likely to occur on the contralateral side [6]. The reason for this is that in an incomplete CoW, other blood vessels must compensate for the blood flow, and the flow velocity in these vessels increases [7]; this increases hemodynamic stress on arterial walls, which is thought to lead to aneurysms and bleeding.

Regarding cerebral infarction, some studies have reported that an incomplete anterior CoW is a risk factor for ipsilateral cerebral infarction [8], while others have found no such association [9]. Thus, the relationship between cerebral infarction and CoW variation remains controversial. However, based on prior findings related to aneurysms and cerebral hemorrhage, an incomplete CoW may cause a difference in blood flow between the left and right sides, which may lead to differences in the incidence of cerebral infarction. While previous studies have primarily focused on whether variations in the Circle of Willis are associated with the risk of stroke occurrence, far fewer have examined their potential role in determining the laterality of infarction within affected patients. In particular, the impact of A1 segment hypoplasia or aplasia on infarct distribution (i.e., dominant vs. minor side) remains insufficiently investigated, and the distinction between stroke susceptibility and infarct laterality has not been clearly addressed in the literature. Furthermore, most prior studies have not incorporated hemodynamic asymmetry based on A1 dominance, nor have they examined these effects across different etiological subtypes of stroke.

Therefore, our study uniquely focuses on the relationship between A1 segment hypoplasia/aplasia and infarct laterality, taking into account both A1 dominance and stroke etiology (lacunar, CES, and ESUS).

## 2. Methods

We retrospectively reviewed our single institutional database of consecutively hospitalized patients with stroke aged 18 years or older between April 2017 and March 2023 and enrolled those with unilateral anterior circulation cerebral infarction during the study period. We classified the type of cerebral infarction based on the Trials of Org 10172 in Acute Stroke Treatment criteria. Among those classified as cerebral infarction of undetermined etiology, we diagnosed embolic stroke of undetermined source (ESUS) according to the diagnostic criteria of the Cryptogenic Stroke/ESUS International Working Group. Next, we compared patient background characteristics for each A1 classification, lacunar infarction, cardiogenic cerebral embolism (CES), and ESUS. Patients with large-artery atherosclerotic infarction were excluded because it is difficult to distinguish A1 hypoplasia from atherosclerotic stenosis using magnetic resonance angiography (MRA). Each CoW was evaluated using MRA. MRA was performed using a three-dimensional time-of-flight (3D TOF) technique. The A1 segment was evaluated on maximum intensity projection (MIP) images and source images. The diameter of the A1 segment was assessed at its proximal portion. Images were reviewed on a clinical workstation using the institutional PACS viewer.

A1 hypoplasia was defined as a maximum A1 diameter of <1 mm on MRA for the purposes of this study. This threshold was selected based on practical considerations and should be regarded as an operational definition, as no universally accepted cutoff exists, and A1 aplasia was defined as A1 not being visually identified on MRA.

**Figure 1:**
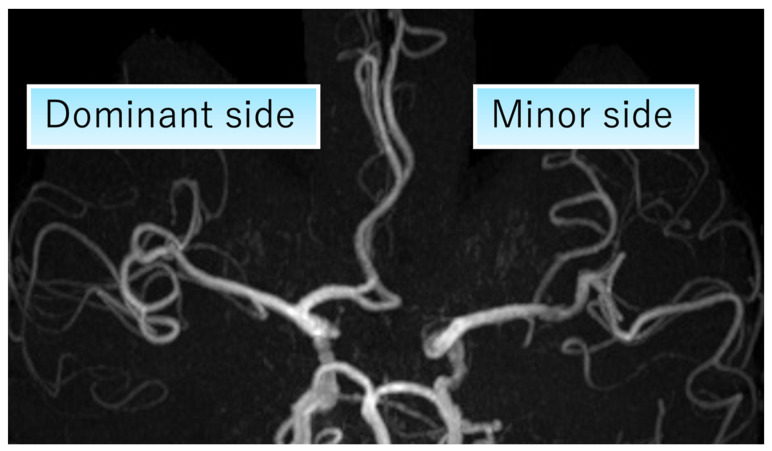
The minor side was defined as the side with A1 hypoplasia or aplasia, and the dominant side as the contralateral side.

We defined the side with A1 hypoplasia or aplasia as the minor side, and the contralateral side as the dominant side (Figure 1) and evaluated whether cerebral infarction occurred on the minor or dominant side. All MRA images were reviewed by a neurologist specializing in stroke who was blinded to the clinical information, including stroke subtype and infarct laterality.

We also collected patient medical histories, including hypertension, diabetes, dyslipidemia, smoking status, and atrial fibrillation. We analyzed the proportion of patients with A1 hypoplasia or aplasia according to the cerebral infarction type.

This study was conducted and reported in accordance with the STROBE (Strengthening the Reporting of Observational Studies in Epidemiology) guidelines.

## 3. Statistical Analysis

Categorical variables are presented as numbers (percentages), and continuous variables are presented as mean ± standard deviation. Univariate comparisons between categorical variables were performed using the chi-square test, Fisher’s exact test, and unpaired t-test. All statistical tests were two-sided, with *p*-values < 0.05 used to denote statistical significance. Confidence intervals (95%) for proportions were calculated using the Wilson method to estimate the precision of the observed proportions.

No formal sample size calculation was performed, as this was a retrospective exploratory study based on consecutively enrolled patients during the study period, and the results should be interpreted as hypothesis-generating. Multivariable analysis was not performed due to the limited sample size and the small number of events in each subgroup.

## 4. Results

**Figure 2:**
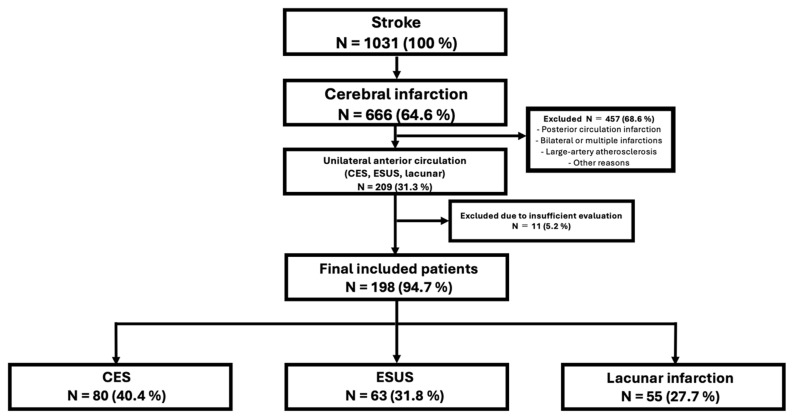
Patient selection flowchart. Percentages are calculated based on the number of patients at each stage. Patients were excluded according to the predefined eligibility criteria described in the Methods, including non-anterior circulation infarction, bilateral or multiple infarctions, and large-artery atherosclerotic infarction.

Overall, 1031 patients with stroke were hospitalized during the study period. Among them, 666 had a cerebral infarction. Of the 209 patients with unilateral anterior circulation cerebral infarction, 55 had lacunar infarction, 89 had CES, and 65 had ESUS. Among the 65 patients with ESUS, two were excluded because the MRA evaluation was insufficient due to motion artifacts, and the remaining 63 were enrolled. Of the 89 patients with CES, nine were excluded because of inadequate MRA imaging (two without magnetic resonance imaging [MRI], three with internal carotid artery (ICA) occlusion, and four with motion artifacts), and the remaining 80 were enrolled. Among these 80 patients with CES, the background etiology was atrial fibrillation (AF) in 76 patients, cardiac tumor in one, thrombus in a ventricular aneurysm in one, severe heart failure in one, and artificial valve in one, with AF accounting for most cases. A flowchart is shown in Figure 2.

The prevalence of A1 hypoplasia or aplasia was also evaluated. Among the 198 registered patients, 61 (30%) had A1 hypoplasia or aplasia. Of these 61 patients, 40 (65%) had A1 hypoplasia or aplasia on the left side, with the right side being the dominant side. No cases of bilateral A1 hypoplasia were observed. We compared patient backgrounds (hypertension, dyslipidemia, and diabetes) between those with complete A1 segment and those with A1 hypoplasia or aplasia but found no significant differences (Table 1).

When evaluated according to the cerebral infarction type, A1 hypoplasia or aplasia was present in 17 of 55 (31%) patients with lacunar infarction, 24 of 80 (30%) with CES, and 20 of 63 (31%) with ESUS, with no significant difference in the prevalence of A1 hypoplasia among the cerebral infarction types (chi-square test; *p* = 0.98; Table 2).

Next, we evaluated the association between the cerebral infarction site and A1 aplasia or hypoplasia. Among patients with A1 aplasia or hypoplasia, the proportion of those who developed cerebral infarction with A1 dominance was investigated. Nine of 17 (53%) patients had lacunar infarction, 11 of 20 (55%) had ESUS, and 18 of 24 (75%) with CES had cerebral infarction on the A1 dominant side (Table 3). No statistically significant difference was observed (Yates’ chi-square test; *p* = 0.43). Any observed differences in subgroup analyses, including the CES group, should be interpreted with caution given the limited sample size.

We examined whether the side most likely to develop cerebral infarction in CES coincided with the side most likely to exhibit A1 dominance. When examining the distribution of A1 dominant sides in patients with CES, 17 patients (71%) had left-side dominance, and seven (29%) had right-side dominance, indicating a higher prevalence of left-side dominance.

Among 56 patients with CES with complete anterior circulation (absence of A1 aplasia or hypoplasia), cerebral infarction was distributed on the left side in 30 (54%) and on the right side in 26 (46%). Among 18 patients with CES with left A1 dominance, cerebral infarction occurred on the left side in 14 patients (76%) and on the right side in four (24%). Among seven patients with right A1 dominance, cerebral infarction occurred on the left side in two patients (29%) and on the right side in five (71%) (Table 4). Although differences in proportions were observed in the CES subgroup, these were not statistically significant and should be interpreted with caution given the limited sample size.

## 5. Discussion

This study did not identify a statistically significant association between A1 hypoplasia/aplasia and infarct laterality. The findings remain inconclusive, including in subgroup analyses such as the CES group, given the limited sample size.

Although the reported prevalence of A1 hypoplasia or aplasia varies slightly depending on patient backgrounds and the imaging modality used, previous studies have reported that the prevalence of A1 aplasia ranges from 5.6% to 6.7%, whereas that of A1 hypoplasia ranges from 10% to 35% [10,11,12]. In this study, the prevalence of A1 aplasia or hypoplasia was 30%, consistent with these previous reports.

The relationship between A1 aplasia or hypoplasia and cerebral infarction remains controversial. Previous reports have shown that the incidence of A1 hypoplasia is higher in patients with cerebral infarction than in healthy controls, suggesting that impaired collateral circulation may be involved in the onset of cerebral infarction, with striatal lacunar infarction particularly common on the A1 hypoplasia side [13]. In contrast, other studies have reported no association between A1 hypoplasia and cerebral infarction. Shaban et al. reported no association between A1 hypoplasia or aplasia and the incidence, volume, or severity of cerebral infarction [14]. Oumer et al. conducted a systematic review to evaluate the association between CoW variations, including A1 aplasia or hypoplasia, and cerebral infarction but found no association [9].

In the present study, there was no difference in the prevalence of A1 aplasia or hypoplasia across different cerebral infarction types, suggesting that A1 aplasia or hypoplasia is unlikely to be a risk factor for specific cerebral infarction types. No statistically significant association was identified. The findings remain inconclusive given the limited sample size. Therefore, the results should be interpreted as exploratory and hypothesis-generating, and any proposed pathophysiological explanations remain speculative.

A possible hemodynamic explanation may be considered, whereby differences in carotid blood flow between the dominant and minor sides could influence embolic distribution. Previous studies have suggested that blood flow may be relatively higher on the dominant side [15,16]; however, this interpretation remains speculative and was not directly evaluated in the present study.

Previous studies on the left–right distribution of CES have reported inconsistent findings, with no clear pattern established [17,18,19]. This suggests that multiple factors may be involved. Our findings may be associated with differences in blood flow between the dominant and minor sides; however, this remains speculative and was not directly evaluated in the present study.

In this study, unlike CES, no difference was found in the incidence of cerebral infarction between the A1 dominant and minor sides in patients with ESUS. Initially, the primary embolic source in ESUS was occult paroxysmal atrial fibrillation (PAF). However, in the CRYSTAL-AF trial, which evaluated the detection rate of PAF using an insertable cardiac monitor in patients with cryptogenic stroke, the detection rate was only 12.4% at 12 months [20]. Furthermore, two randomized controlled trials investigating the effectiveness of direct oral anticoagulants (DOACs) in ESUS were unable to demonstrate the effectiveness of DOACs for any antiplatelet agent [21,22], suggesting that the proportion of PAF may be low in patients with ESUS. Based on these findings, it is speculated that ESUS encompasses a range of pathologies, such as non-stenotic carotid artery plaque, aortogenic embolism, and paradoxical cerebral embolism. If the pathology of ESUS is a carotid artery lesion, such as a carotid web or a small, non-stenotic carotid artery plaque, cerebral embolism will occur on the side of the carotid artery lesion, regardless of the difference in carotid artery blood flow between the left and right sides. These findings may be consistent with the absence of a statistically significant difference in the occurrence of cerebral embolism between the A1 dominant and minor sides in ESUS.

The absence of statistical significance in this study should not be interpreted as evidence of no association. However, given the limited sample size and number of events, the study was underpowered to detect modest differences, and the findings do not allow for a reliable conclusion regarding the presence or absence of a true association. Therefore, the results should be considered exploratory.

Our study has some limitations. First, the retrospective design may have introduced systematic selection bias; thus, it cannot be used to prove causality. Second, it is difficult to completely distinguish between A1 aplasia or hypoplasia and A1 atherosclerotic stenosis using MRA; therefore, it is possible that some cases diagnosed as A1 aplasia or hypoplasia included those with atherosclerotic stenosis. Evaluation of MRI plaque images may be useful for distinguishing between these conditions. Third, the imaging evaluation was performed by a single reviewer, and interobserver variability was not assessed, which may have introduced potential bias in image interpretation. This may also limit the consistency of the anatomical classification. Therefore, the findings should be interpreted with caution. Fourth, the relatively small sample size limited the statistical power and precluded multivariable analysis due to the small number of events in each subgroup, increasing the risk of model overfitting. Because multivariable analysis was not performed, the observed association cannot be interpreted as independent of potential confounding factors. In addition, the small size of each subgroup may further limit the robustness of the findings. Therefore, the present analysis should be interpreted as exploratory. Finally, this was a single-center study, which may limit the generalizability of the findings.

## 6. Conclusions

In conclusion, no statistically significant association was identified. The findings remain inconclusive and do not allow for causal or independent inferences. Therefore, the results should be considered exploratory and hypothesis-generating.

## Figures and Tables

**Table 1:** Comparison of background characteristics between patients with complete A1 segment and those with A1 hypoplasia.

	Complete A1 (n = 137)	A1 Hypoplasia/Aplasia (n = 61)	*p*-Value
Male, n (%)	79 (58)	26 (43)	0.05
Age (mean ± SD) years	72.1 ± 12.0	73.0 ± 13.7	0.67
NIHSS (mean ± SD) Points	6.8 ± 9.0	6.1 ± 8.0	0.63
Hypertension, n (%)	87 (63)	40 (65)	0.77
Dyslipidemia, n (%)	61 (44)	28 (45)	0.85
Diabetes, n (%)	38 (27)	14 (22)	0.47
AF, n (%)	47 (34)	25 (40)	0.36
CKD, n (%)	22 (16)	11 (18)	0.73
Dialysis, n (%)	9 (6)	6 (9)	0.42
Smoker, n (%)	42 (30)	17 (27)	0.69
SAS, n (%)	6 (4)	1 (1)	0.58

AF, atrial fibrillation; CKD, chronic kidney disease; NIHSS, National Institutes of Health Stroke Scale; SAS, sleep apnea syndrome; SD, standard deviation.

**Table 2:** Prevalence of A1 hypoplasia or aplasia by cerebral infarction type.

Infarction Type	Complete A1	A1 Hypoplasia or Aplasia
Lacunar, n (%)	38 (69)	17 (31)
ESUS, n (%)	43 (69)	20 (31)
CES, n (%)	56 (70)	24 (30)

ESUS, embolic stroke of undetermined source; CES, cardiogenic cerebral embolism.

**Table 3:** Comparison of infarction side in patients with A1 hypoplasia or aplasia, based on the infarction type.

Infarction Type	Infarction on A1 Dominant Side	Infarction on A1 Minor Side
Lacunar, n (%)	9 (53%; 95% CI: 26–79)	8 (47%; 95% CI: 20–73)
ESUS, n (%)	11 (55%; 95% CI: 31–78)	9 (45%; 95% CI: 21–68)
CES, n (%)	18 (75%; 95% CI: 56–94)	6 (25%; 95% CI: 6–43)

ESUS, embolic stroke of undetermined source; CES, cardiogenic cerebral embolism.

**Table 4:** Left–right distribution of cerebral infarction in patients with CES according to A1 dominance type.

A1 Type	Left-Sided Infarction	Right-Sided Infarction
Complete A1, n (%)	30 (54)	26 (46)
Left A1 dominant, n (%)	14 (76)	4 (24)
Right A1 dominant, n (%)	2 (29)	5 (71)

CES, cardiogenic cerebral embolism.

## Data Availability

The datasets generated and/or analyzed during the current study are not publicly available due to privacy and ethical restrictions but are available from the corresponding author on reasonable request.

## Abbreviations

AF, atrial fibrillation; CES, cardioembolic stroke; CoW, Circle of Willis; DOAC, direct oral anticoagulant; ESUS, embolic stroke of undetermined source; ICA, internal carotid artery; MRA, magnetic resonance angiography; MRI, magnetic resonance imaging.
